# Book Notices

**Published:** 1897-01

**Authors:** 


### Book Notices.

---

ANNOUNCEMENT.—E. B. Treat, publisher, New York, has in press for issuance early in 1897, the International Medical Annual; being the fifteenth yearly issue of that well-known one-volume reference work. The prospectus shows that the volume will be the result of the labors of upwards of forty physicians and surgeons, of international reputation, and will present the world's progress in medical science.

The publisher states that the kind reception accorded to the Medical Annual has rendered it possible for him to spare no expense in its production; while the editorial staff have devoted a large amount of time and labor in so condensing the literary matter, as to confine the volume within a reasonable size, without omitting facts of practical importance.

The value of the work will be greatly enhanced by the thoroughness of illustration, both colored plates and photographic reproductions in black and white will be used wherever helpful in elucidating the text.

"To those who need the condensed and well-arranged presentation of the medical advances of the past year—and this class must necessarily include all physicians—we heartily commend the International Medical Annual."

The volume will contain about 700 pages. The price will be the same as heretofore, $2.75. Full descriptive circular will be sent upon application to the publisher.

---

Twentieth Century Practice.—An international encyclopedia of modern medical science. By leading authorities of Europe and America. Edited by Thomas L. Stedman, M. D., New York City. In twenty volumes, Vol. VII. "Diseases of the Respiratory Organs and Blood, and Functional Sexual Disorders." New York: William Wood and Company. 1896.

With the publication of this volume, the series of the "Twentieth Century Practice" is completed as far as and including Vol. VIII., it having been necessary to publish the eighth before the seventh volume. This volume is the work of ten contributors, seven of whom are Americans.

The different diseases here discussed are treated of in an exhaustive way, just as all subjects appearing in the preceding volumes have been.

The first article is by Dr. Herbert B. Whitney, of Denver, on diseases of the pleura. Dr. Franz Riegel, of Giessen, discusses asthma; and Dr. E. Fletcher Ingals, of Chicago, hay fever. Diseases of the mediastinum, and of the diaphragm, are treated of by Dr. E. Main, of Paris. Dr. Alfred Stengel, of Philadelphia, contributes an exhaustive article on diseases of the blood. This article covers 195 pages, and includes a discussion of plethora, oligæmia, hydræmia, anhydræmia, coagulation, lipæmia, melanæmia, leucocytosis, hypoleucocytosis, polycythæmia, anæmia, symptomatic anæmia, chlorosis, progressive pernicious anæmia, leukæmia, Hodgkin's disease, anæmia infantum pseudo-leukæmica, hœmocytolysis, hœmoglobinæmia, paroxysmal hæmoglobinuria, purpura, purpura simplex, purpura rheumatica, purpura-hæmorrhagica, scurvy, scurvy in infancy, hæ-

mophilia, etc. Dr. Jules Comby, of Paris, writes on rachitis, and Drs. Ernest M. Cushing and Charles G. Cumston, both of Boston, on the disorders of menstruation. A chapter on functional diseases of the male sexual organs, by Dr. Charles W. Allen, of New York, and one on the chemical and microscopical examination of the urine, by Dr. James M. French, of Cincinnati, complete the volume.

The entire volume covers nearly 800 pages, and for the general practitioner, is one of the best yet issued of this great work. The subjects are all important ones, and the practical and thorough way in which they are handled by the different contributors commends the volume to the profession, making it almost indispensable. S. E. H.

---

Over the Hookah, The Tales of a Talkative Doctor: By G. Frank Lydston, M. D., Professor of Genito-Urinary Surgery in the Chicago College of Physicians and Surgeons. Professor of Criminal Anthropology in the Kent College of Law, etc. Sold by subscription only. Sent prepaid on receipt of subscription price. Price in cloth, gilt top, $4.00. Price in morocco, full gilt, $5.00. Over 600 pages octavo. Profusely illustrated from the author's designs by C. Everett Johnson. The Fred Klein Publishing Co., Chicago.

This is, indeed, a rare book. It must be read to be appreciated. No words can do justice to the sparkling wit, the keen satire, mostly aimed at ye doctor, the gruesome humor with which every page abounds. It is not, though a funny book, by any means; it may be said to be humorous, but not funny. Dr. Lydston has put into the mouth of his leading character, the spokesman, Dr. Weymouth, who, nightly, unloads it to a young medical student, who being under obligations—and the sole auditor, cannot well do otherwise but submit to it, a vast amount of anecdote, reminiscence and experiences, covering a vast range of subjects,—enough to fill, as it does, a large book. It is, for the most part interesting, a little prolix in some places, the agony too long drawn out; but in its entirety, it is very readable. The worth of the book as a whole, is somewhat marred by the introduction of dialect stories. As great as Dr. Lydson's versatility is—and, really one should not be expected to excell in everything, dialect story-telling, negro, Irish and the new style—the Bowery, or Chimmy Fadden lingo—is not his fort—though he acquits himself fairly well. His success in that line would doubtless have been better had he exercised a little discrimination in his selection, and left out, especially, the Kentucky story of the mint julips, a yarn that had whiskers tinged with the frosts of time, when Noah was a kid.

Dr. Lydston is an ardent worshipper at the shrine of Nicotiana. Every chapter is preceded by an invocation to the pipe, like grace before meat.

If any one part is better than other, it is the "Rhodomontade of a Skull"; it is sparkling, and discounts Munschausen's best.

The book is beautifully illustrated. The designs were drawn, we understand, by the talented author, and do him credit. The book ought to have a wide sale amongst the doctors, and we venture the belief that it will. We observe with some satisfaction that the doctor, who has been, in this respect, an eclectic, has incorporated in his book—the Red Back's Dr. Merriman's joke on Dr. Daniel and Dr. Thompson; the Sunflower story.

D.

---

The Ready-Reference Handbook of Diseases of the Skin By George Thomas Jackson, M. D., (Col.), Professor of Dermatology in the Woman's Medical College of the New York Infirmary, and in the Medical Department of the University of Vermont, etc., etc. Second edition, revised and enlarged—594 pages, 69 illustrations. Price in cloth, $2.75. Lea Brothers & Co., Publishers, New York and Philadelphia, 1896.

The first edition of this work was published in 1892, and was so favorably received by the profession that a second has become necessary. In this edition the author has, besides thoroughly revising the first one, so as to bring it down to the present date, added new sections on a number of subjects, such as Acromegaly, Actinomycosis, Angioma derpiginosum, Baelzer's Disease, Cheilitis glandularis, Clavus syphiliticus, Dermatitis repens, etc. Nineteen new illustrations have been inserted, and the text has been considerably increased. The arrangement of the subjects is alphabetical, making the book more convenient for reference. We take pleasure in commending this handbook to the student and the medical practitioner. Both will be greatly benefited by studying it carefully.

